# Causal Effects of the Affordable Care Act (ACA) Implementation on Non-Hodgkin's Lymphoma Survival: A Difference-in-Differences Analysis

**DOI:** 10.7759/cureus.52571

**Published:** 2024-01-19

**Authors:** Oluwasegun A Akinyemi, Terhas Asfiha Weldeslase, Mojisola E Fasokun, Eunice Odusanya, Eunice O Mejulu, Ejura Y Salihu, Ngozi T Akueme, Kakra Hughes, Miriam Micheal

**Affiliations:** 1 Health Policy and Management, University of Maryland School of Public Health, College Park, USA; 2 Surgery, Howard University, Washington DC, USA; 3 Surgery, Howard University College of Medicine, Washington, USA; 4 Epidemiology and Public Health, University of Alabama at Birmingham, Birmingham, USA; 5 Obstetrics and Gynecology, Howard University College of Medicine, Washington DC, USA; 6 Medical School, Western Illinois University, Illinois, USA; 7 Department of Health Services Research, University of Wisconsin, Madison, USA; 8 Dermatology, University of Medical Sciences (UNIMED), Ondo State, NGA; 9 Surgery, Howard University College of Medicine, Washington DC, USA; 10 Internal Medicine, Howard University College of Medicine, Washingon DC, USA; 11 Internal Medicine, University of Maryland School of Medicine, Baltimore, USA

**Keywords:** cancer-specific survival, health policy, affordable care act, difference in difference, non-hodgkin's lymphoma

## Abstract

Introduction: Non-Hodgkin's Lymphoma (NHL) accounts for a substantial number of cancer cases in the United States, with a significant prevalence and mortality rate. The implementation of the Affordable Care Act (ACA) has the potential to impact cancer-specific survival among NHL patients by improving access to healthcare services and treatments.

Objective: This study aims to assess the impact of the implementation of the ACA on cancer-specific survival among patients diagnosed with NHL.

Methodology: In this retrospective analysis, we leveraged data from the Surveillance, Epidemiology, and End Results (SEER) registry to assess the impact of the ACA on cancer-specific survival among NHL patients. The study covered the years 2000-2020, divided into pre-ACA (2000-2013) and post-ACA (2017-2020) periods, with a three-year washout (2014-2016). Using a Difference-in-Differences approach, we compared Georgia (a non-expansion state) to New Jersey (an expansion state since 2014). We adjusted for patient demographics, income, metropolitan status, disease stage, and treatment modalities.

Results: Among 74,762 patients, 56.2% were in New Jersey (42,005), while 43.8% were in Georgia (32,757). The pre-ACA period included 32,851 patients (51.7% in Georgia and 56.7% in New Jersey), and 27,447 patients were in the post-ACA period (48.3% in Georgia and 43.4% in New Jersey). The post-ACA period exhibited a 34% survival improvement (OR=0.66, 95% CI 0.58-0.75). ACA implementation was associated with a 16% survival boost among NHL patients in New Jersey (OR=0.84, 95% CI 0.74-0.95). Other factors linked to improved survival included surgery (OR=0.86, 95% CI 0.81-0.91), radiotherapy (OR=0.77, 95% CI 0.72-0.82), and married status (OR=0.67, 95% CI 0.64-0.71).

Conclusion: The study underscores the ACA's potential positive impact on cancer-specific survival among NHL patients, emphasizing the importance of healthcare policy interventions in improving patient outcomes.

## Introduction

Non-Hodgkin's Lymphoma (NHL) represents a significant portion of cancer cases in the United States, accounting for 4% of all cancers, with its prevalence and mortality rates posing a substantial public health concern [1]. This group of diverse lymphoid cancers is characterized by varying prognoses and clinical presentations, making its management complex and multifaceted [2]. The role of healthcare accessibility in managing NHL cannot be overstated [3], especially in a country like the United States, where healthcare policies and insurance coverage significantly influence patient outcomes.

The introduction of the Affordable Care Act (ACA) in 2010 marked a pivotal moment in U.S. healthcare policy, aiming to increase insurance coverage and accessibility to healthcare services [4-6]. This legislation was expected to have far-reaching implications on the management and outcomes of chronic and severe conditions like cancer. For diseases like NHL, where timely diagnosis and access to comprehensive treatment are crucial, the ACA's impact on patient survival rates is of great interest. However, the extent to which ACA implementation has influenced cancer-specific outcomes, especially for the NHL, remains an area needing in-depth exploration.

This study, therefore, aims to bridge this gap by analyzing the effects of ACA implementation on the survival rates of NHL patients. By examining the differences in patient outcomes between states with varying levels of ACA implementation, this research seeks to provide empirical evidence on the ACA's impact in the context of cancer care. Specifically, this retrospective analysis focuses on comparing two U.S. states, Georgia and New Jersey, to assess the impact of the ACA on NHL survival rates using a Difference-in-Differences approach.

Georgia, which did not implement the ACA, and New Jersey, which implemented the policy in 2014, present a unique opportunity to understand how differences in healthcare policy implementation can influence cancer survival rates. The study period spans two decades (2000-2020), divided into pre-ACA (2000-2013) and post-ACA (2017-2020) phases, with a three-year washout period (2014-2016) to account for the transitional phase of ACA implementation [7-11].

Through this analysis, we aim to contribute to the ongoing discourse on the efficacy of healthcare policies like the ACA in improving health outcomes, particularly in the context of cancer care. This study is poised to offer valuable insights into how policy interventions can be leveraged to enhance patient survival rates, thereby informing future healthcare policy decisions and strategies for managing complex diseases such as NHL.

## Materials and methods

Dataset

This study utilized data from the Surveillance, Epidemiology, and End Results (SEER) Program registry. SEER provides comprehensive information on cancer incidence and survival in the United States and is a reliable source for longitudinal cancer data. The SEER registry gathers cancer incidence data from cancer registries representing about 50% of the U.S. population. Since its inception on January 1, 1973, the SEER database has collected cancer case data. Initially, it covered Connecticut, Iowa, New Mexico, Utah, Hawaii, and the metropolitan areas of Detroit and San Francisco-Oakland. Over time, the SEER Program has expanded to include many additional regions. It has nine registries covering Kentucky, Greater California, Utah, Louisiana, Georgia, New York, Massachusetts, Wisconsin, and Idaho. The funding is a collaborative effort involving the National Cancer Institute (NCI), the Centers for Disease Control and Prevention (CDC) through the National Program of Cancer Registries, as well as with state-level funding. These registries provide information on patient demographics, the location and type of the primary tumor, the stage of cancer at diagnosis, and initial treatment approaches, and they continuously track patient survival status. The dataset included records from 1975 to 2020.

For this retrospective study, we analyzed publicly available de-identified datasets to ensure confidentiality and privacy. Given the use of publicly accessible, non-personal data, Institutional Review Board (IRB) approval was not required for this study.

Study population

The study population comprised patients diagnosed with NHL from 2000 to 2020. We included cases from New Jersey, a state that had adopted the ACA, and Georgia, which is yet to implement the policy as of 2023. Other states in the SEER registry that have implemented the ACA include California, New Jersey, Hawaii, Connecticut, Iowa, Kentucky, Louisiana, New Mexico, Seattle, and Utah. As of 2023, Georgia is the only state in the SEER registry that has not yet implemented the ACA. This design allowed for a comparative analysis between the two groups.

Outcome variables

The primary outcome variable was cancer-specific survival, measured from the date of diagnosis to the date of death due to NHL. In the SEER database, a specific variable is dedicated to recording the causes of death. This variable not only tracks cancer-specific mortality but also captures deaths due to other causes in patients diagnosed with cancer. Survival time was censored at the end of the study period or at the time of death due to causes. The survival time in months was further converted into years for more helpful data handling.

Inclusion/exclusion criteria

We defined specific inclusion and exclusion criteria for participant selection. The inclusion criteria comprised patients with a confirmed diagnosis of NHL and an age range at diagnosis from 18 to 85 years. Conversely, we excluded participants with a prior history of any cancer and those with incomplete records pertaining to survival status or essential demographic information. We also excluded patients with missing data on disease staging at the presentation since this is a very important variable in cancer-specific survival analyses.

Covariates/independent variables

Covariates included in the analyses include a range of patient demographics such as age and race/ethnicity. Age was a continuous variable that ranged from 18-85 years. This was further categorized into groups 18-44 years, 45-64 years, and ≥ 65 years. The patient’s race/ethnicity was categorized as non-Hispanic White (White), non-Hispanic Black (Black), Hispanic, and non-Hispanic Other (Other). We also consider socioeconomic factors, specifically income levels, to understand their impact. Our analysis used the median household income based on patients' ZIP Codes for 2019, traditionally used by SEER. We stratified the household incomes into four quartiles: Quartile I (ranging from <$35,00 to $49,999), Quartile II ($50,000 to $64,999), Quartile III ($65,000 to $74,999), and Quartile IV ($75,000 and above). The SEER database removed insurance status in the more recent release of the database; therefore, we only utilized the median household income as a proxy to measure socioeconomic status. Additionally, we account for metropolitan status by differentiating between urban and rural residences. Disease stage at diagnosis is another crucial variable, as it provides insight into the severity of the condition at the time of detection. The disease stage at presentation was classified as stage I, II, III, and IV. Finally, the treatment modalities, including surgery, chemotherapy, and radiation, are included to evaluate their influence on patient outcomes. These covariates collectively contribute to a comprehensive understanding of the study's scope. A significant limitation of the SEER database is the absence of measures of preexisting comorbidities such as hypertension, diabetes, obesity, etc.

The difference-in-differences methodology

We employed a difference-in-differences (DID) analysis to estimate the causal impact of the implementation of the ACA on survival following a diagnosis of NHL. This method compared the change in survival rates over time between New Jersey, a state that implemented the ACA, and Georgia, which did not. Pre-ACA (2000-2013) and post-ACA (2017-2020) periods were analyzed, with a three-year washout period (2014-2016) to provide sufficient time for the ACA's policies to be fully implemented in New Jersey, which implemented the ACA in January 2014. and their effects to become evident. The DID approach allowed us to compare the cancer-specific survival of patients with NHL before and after the implementation of the policy. In the DID analysis, we identified the 'treated' group (New Jersey) and a comparable 'control' group, in this case, Georgia, that has not implemented the policy as of 2023. This comparison enabled us to isolate and estimate the effect of the ACA policy on cancer-specific survival. 

Sensitivity analysis

Sensitivity analyses were conducted to test the robustness of the findings. This included using alternative definitions of the treatment group (states implementing ACA) and varying the length of the washout period. Additionally, analyses were repeated, excluding patients with late-stage diagnoses, to assess the impact of the ACA on early-stage cancer survival.

Statistical analysis

Statistical analyses were performed using STATA software (StataCorp LLC., College Station, TX). Kaplan-Meier survival curves were generated, and log-rank tests were used to compare survival distributions between the ‘treated’ and the control group. Cox proportional hazard models were used to estimate hazard ratios (HRs) for death, adjusting for patient demographics, disease stage, income, marital status, and residence status, as well as treatment modalities like surgery, radiotherapy, and chemotherapy. The parallel trends assumption was verified using graphical methods and statistical tests. Results were considered statistically significant at a p-value < 0.05.

## Results

In the present study spanning 2000-2020, significant disparities were observed in the baseline characteristics between New Jersey and Georgia populations (Table 1). In New Jersey, a higher proportion of the population was aged ≥ 65 years (56.0%) compared to Georgia (50.4%), with this age discrepancy being statistically significant (p < 0.001). Female representation was marginally higher in Georgia (54.8%) than in New Jersey (53.4%, p = 0.001). Racial and ethnic differences were prominent, notably with more Black individuals in Georgia (21.0% vs. 8.0% in New Jersey, p < 0.01). Substantial variations were also evident in the two states' healthcare access dynamics (pre- and post-ACA), marital status, urban versus rural residence, household income levels, and cancer. Furthermore, treatment approaches, including chemotherapy, radiotherapy, and surgery, showed significant differences (all p < 0.001). These findings highlight critical regional variations in demographic and healthcare-related factors.

**Table 1:** Baseline Characteristics of Study Population (SEER Registry 2000-2020) ACA, Affordable Care Act, Yrs., years, p < 0.001, Statistical significance

Variable	Total (N=74,762)	New Jersey (n=42,005)	Georgia (n=32,757)	p-value
Age				<0.001
< 45 Yrs.	9,092 (12.5%)	4,589 (11.2%)	4,503 (14.0%)	
45-64 Yrs.	24,829 (34.0%)	13,387 (32.8%)	11,442 (35.6%)	
≥ 65 Yrs.	39,049 (53.5%)	22,855 (56.0%)	16,194 (50.4%)	
Female	40,376 (54.0%)	22,419 (53.4%)	17,957 (54.8%)	0.001
ACA (ACA Periods)				<0.001
Pre-ACA	32,851 (54.5%)	19,230 (56.7%)	13,621(51.7%)	
Post-ACA	27,447 (45.5%)	14,718 (43.4%)	12,729 (48.3%)	
Race/Ethnicity				<0.001
White	56,569 (76.0%)	32,549(78.0%)	24,020 (73.5%)	
Black	10,207 (13.7%)	3,336 (8.0%)	6,871 (21.0%)	
Hispanic	5,542 (7.5%)	4,308 (10.3%)	1,234 (3.8%)	
Others	2,082 (2.8%)	1,523 (3.7%)	559 (1.7%)	
Married	39,137 (58.1%)	21,697 (58.6%)	17,440 (57.6%)	< 0.001
Rural/Metro Status				< 0.001
Non-Metro	6,588 (8.8%)	0 (0.0%)	6,588 (20.1%)	
Metro I (Residents ≈ 250K)	6,274 (8.4%)	1,275 (3.0%)	4,999 (15.3%)	
Metro II (Residents ≈ 250K-1M)	7,390 (9.9%)	3,633 (8.7%)	3,757 (11.5%)	
Metro III (Residents > 1M)	54,458 (72.9%)	37,045 (88.3%)	17,413 (53.2%)	
Median Household Income				<0.001
Quartile I	8, 477 (11.4%)	0 (0.0%)	8,477 (25.9%)	
Quartile II	6,090 (8.2%)	343 (0.8%)	5,747 (17.5%)	
Quartile III	21,191 (28.4%)	11,657 (27.8%)	9,534 (29.1%)	
Quartile IV	38,952 (52.1%)	29,953 (71.4%)	8,999 (27.5%)	
Disease stage at Presentation Stage				<0.001
Stage I	17,867 (49.6%)	9,651 (50.4%)	8,216 (48.7%)	
Stage II	9,937 (27.6%)	5,317 (27.8%)	4,620 (27.4%)	
Stage III	2,833 (7.9%)	1,408 (7.4%)	1,425 (8.4%)	
Stage IV	5,392 (15.0%)	2,768 (14.5%)	2,624 (15.5%)	
Treatments				
Chemotherapy	39,995 (53.5%)	21,556 (51.3%)	18,439 (56.3%)	<0.001
Radiotherapy	10,875 (14.7%)	5,998 (14.3%)	4,877 (15.1%)	0.06
Surgery	18,083 (25.5%)	10,655 (27.4%)	7,428 (23.3%)	<0.001

**Figure 1:**
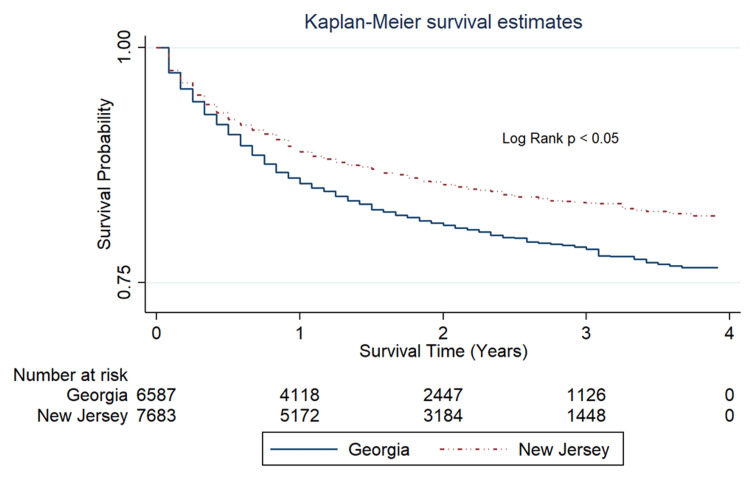
Kaplan-Meier Survival Curve Comparative survival probability of Non-Hodgkin's Lymphoma (NHL) patients in Georgia vs. New Jersey, post-ACA implementation (2017-2020).

Figure 1 presents a Kaplan-Meier survival curve, accompanied by an inserted lifetable, which illustrates the survival probability among patients with non-Hodgkin lymphoma in Georgia and New Jersey during the post-ACA implementation period from 2014 to 2020. This graphical representation provides a comparative analysis of survival rates in these two states following the implementation of the ACA.

Impact of ACA and state expansion

**Figure 2:**
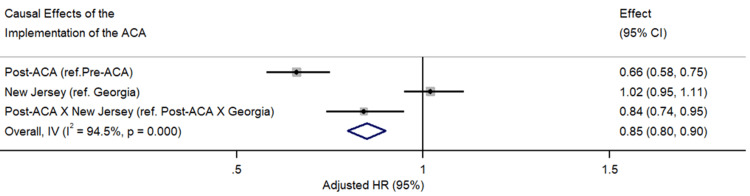
Causal Effects of Implementation of the Affordable Care Act on NHL Survival (SEER Registry 2000-2020)

Post-ACA period: The post-ACA period was associated with a 34% reduction in the mortality hazard (HR = 0.66, 95% CI: 0.58 to 0.75, p < 0.001). While patients in New Jersey, an expansion state, exhibited a marginal hazard of 2% compared to Georgia (HR = 1.02, 95% CI: 0.95 to 1.11, p = 0.617), these differences were not statistically significant. The interaction term for post-ACA in New Jersey showed a 16% reduction in the mortality hazard (HR = 0.84, 95% CI: 0.74 to 0.95, p = 0.007), indicating the potential beneficial impact of ACA implementation in an expansion state (Figure 2).

Impact of treatment modalities and demographics

Table 2 is a Cox regression analysis showing the association of study variables with survival (hazard of death) among patients with NHL. Patients who underwent surgery experienced a 14% reduction in mortality risk (Hazard Ratio (HR) = 0.86, 95% Confidence Interval (CI): 0.81 to 0.91, p < 0.001), while those receiving radiotherapy showed a 23% decrease in risk (HR = 0.77, 95% CI: 0.72 to 0.82, p 0.001). Conversely, chemotherapy was associated with a 34% increase in mortality risk (HR = 1.34, 95% CI: 1.26 to 1.42, p < 0.001). Notable racial disparities were also observed; compared to White patients, Black patients had a 28% higher mortality risk (HR = 1.28, 95% CI: 1.18 to 1.39, p = <0.001), and Hispanic patients had a 7% increase (HR = 1.07, 95% CI: 0.96 to 1.20, p = 0.211). Marital status also played a role, with married patients showing a 33% reduction in mortality risk (HR = 0.67, 95% CI: 0.64 to 0.71, p < 0.001) (Table 2).

**Table 2:** Predictors of Cancer-Specific Survival (SEER Registry 2000-2020) K, thousand, M, million, p < 0.001, statistical significance

Variable	Adjusted HR	95% Confidence Interval		p-value
AGE				
< 45Yrs	Reference	Reference	Reference	Reference
45-64 Yrs	1.64	1.47	1.82	< 0.001
≥ 65 Yrs.	3.157	3.22	3.95	< 0.001
Sex				
Male	Reference	Reference	Reference	Reference
Female	1.28	1.21	1.36	<0.001
Race				
White	Reference	Reference	Reference	Reference
Black	1.31	1.2	1.42	< 0.001
Hispanic	1.13	1.01	1.26	0.035
Others	1.24	1.05	1.46	0.01
Marital Status				
Unmarried	Reference	Reference	Reference	Reference
Married	0.67	0.64	0.71	<0.001
Rural/Metro Status	Reference	Reference	Reference	Reference
Metro I (Residents ≈ 250K)	0.96	0.85	1.09	0.508
Metro II (Residents ≈ 250K-1M)	0.97	0.85	1.11	0.646
Metro III (Residents > 1M)	0.89	0.79	1.01	0.078
Household Median Income				
Income I	Reference	Reference	Reference	Reference
Quartile II	0.93	0.82	1.05	0.233
Quartile III	0.95	0.84	1.07	0.391
Quartile IV	0.9	0.79	1.02	0.101
ACA (Periods)				
Pre-ACA	Reference	Reference	Reference	Reference
Post-ACA	0.66	0.58	0.75	< 0.001
ACA Implementation Status				
Georgia	Reference	Reference	Reference	Reference
New Jersey	1.02	0.95	1.11	0.617
Interraction Term ( ACA X Implementation Status)				
Post-ACA#Georgia	Reference	Reference	Reference	Reference
Post-ACA#New Jersey	0.84	0.74	0.95	0.07
Disease Stage at Presentation				
Stage I	Reference	Reference	Reference	Reference
Stage II	1.21	1.14	1.29	<0.001
Stage III	1.65	1.41	1.94	< 0.001
Stage IV	2.31	2.01	2.65	<0.001
Treatments				
Chemotherapy	1.34	1.26	1.42	<0.001
Radiotherapy	0.76	0.72	0.82	<0.001
Surgery	0.86	0.81	0.91	<0.001

## Discussion

Impact of ACA on cancer-specific survival

The results of our study highlight a substantial impact of the implementation of the Affordable Care Act (ACA) on cancer-specific survival among patients with NHL. This is evidenced by the 34.0% reduction in mortality hazards post-ACA and a further 16.0% reduction in the hazards for patients in the ACA expansion state of New Jersey. These findings align with other studies showing improved health outcomes following ACA implementation [11-13]. For instance, a study by Sommers et al. (2017) found that the ACA improved conditions for patients with chronic conditions, including cancer, potentially contributing to better survival rates [14]. Another study by Siegel et al. (2017) reported improvements in cancer survival rates post-ACA, emphasizing the role of early detection and access to care [15].

The association between ACA implementation and improved survival may be attributed to enhanced access to healthcare, including increased insurance coverage, which facilitates earlier diagnosis and timely treatment. The expansion of Medicaid under the ACA particularly benefited low-income individuals, who are often at greater risk of late-stage cancer diagnoses and poor outcomes. Our findings reinforce the notion that policy interventions, such as the ACA, can significantly impact health outcomes by addressing barriers to healthcare access. 

Other variables and their impact on NHL survival

Surgery and radiotherapy: The survival benefit associated with surgery and radiotherapy in our study is consistent with existing literature that underscores the importance of these treatments in managing NHL. The reduced mortality hazard observed in patients receiving these treatments could be attributed to the effectiveness of these modalities in controlling disease progression.

Marital status: The protective effect of marriage on NHL survival observed in our study resonates with the concept of social support playing a crucial role in cancer prognosis [16-18]. Married individuals might benefit from better emotional support and adherence to treatment, leading to improved outcomes. This finding is supported by previous research, including a study by Aizer et al. (2013), which demonstrated the survival advantage of married individuals in cancer outcomes [19].

Race: The increased mortality hazard for Black patients compared to White patients raises concerns about racial disparities in cancer care. This finding aligns with numerous studies highlighting racial inequities in cancer treatment and outcomes [16,20,21]. Possible reasons for these disparities include differences in access to care, treatment options, and socio-economic factors [22,23].

Chemotherapy: The increased hazard associated with chemotherapy could be reflective of more advanced or aggressive disease at presentation, necessitating chemotherapy. This finding warrants further investigation to understand the nuances of chemotherapy use in NHL patients and its implications on survival.

Policy implication

The policy implications arising from this study are profound. The implementation of the ACA has notably enhanced cancer-specific survival among patients with NHL, particularly evident in Medicaid expansion states like New Jersey compared to non-expansion states such as Georgia. This finding underscores the critical role of healthcare accessibility and insurance coverage in patient outcomes. The study also reveals that marital status significantly influences survival, with married patients experiencing lower mortality risks, suggesting the necessity of developing support systems for all NHL patients. Additionally, the effectiveness of treatments like surgery and radiotherapy in reducing mortality risk emphasizes the need for equitable access to these treatments. The study also points to racial disparities in survival, particularly the increased mortality risk among Black patients, calling for targeted policy interventions to address these inequities in cancer care. Overall, these findings advocate for the expansion of healthcare policies similar to the ACA and emphasize addressing factors such as marital status, income, treatment access, and racial disparities to enhance NHL patient outcomes further.

Limitations and future research

While our study provides valuable insights, it is not without limitations. The retrospective nature of the study and reliance on existing datasets might introduce selection bias. Additionally, the generalizability of the findings may be limited to similar healthcare contexts. We also note that comparing New Jersey's enhanced survival rates pre- and post-Affordable Care Act with similar states not implementing the act is challenging, as the number of non-expansion states continues to decrease. Future research should focus on longitudinal studies that can provide more comprehensive insights into the long-term effects of healthcare policies like the ACA on cancer survival. Additionally, exploring the mechanisms behind socio-economic and racial disparities in cancer outcomes remains a crucial area for further investigation.

## Conclusions

This study provides compelling evidence of the positive impact of the Affordable Care Act on cancer-specific survival among non-Hodgkin's Lymphoma patients, particularly in states that have expanded Medicaid. The significant reduction in mortality hazard post-ACA implementation underscores the crucial role of healthcare accessibility and insurance coverage in improving patient outcomes. Additionally, our findings highlight the importance of treatment modalities like surgery and radiotherapy on survival rates. The observed racial disparities in survival outcomes call for targeted efforts to address inequities in cancer care. This study reinforces the value of healthcare policy interventions in enhancing cancer survival. It emphasizes the need for ongoing research to understand further and mitigate disparities in cancer treatment and outcomes.

## Figures and Tables

**Figure 3:**
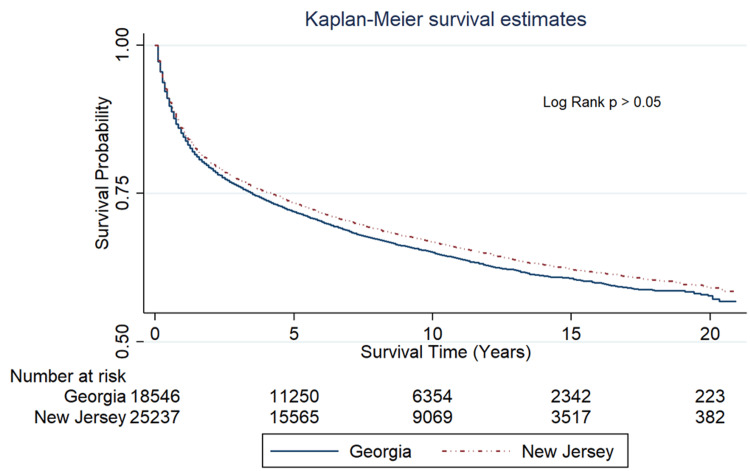
Kaplan-Meier Survival Curve Comparative survival probability of NHL patients in Georgia vs. New Jersey, post-ACA implementation (2000-2013).
